# The addition of STEPPS in the treatment of patients with bipolar disorder and comorbid borderline personality features: a protocol for a randomized controlled trial

**DOI:** 10.1186/1471-244X-14-172

**Published:** 2014-06-09

**Authors:** Georg Riemann, Nadine Weisscher, Peter JJ Goossens, Nel Draijer, Marjolein Apenhorst-Hol, Ralph W Kupka

**Affiliations:** 1 Saxion University of Applied Science, Deventer, The Netherlands; 2 Dimence Mental Health, Center for Bipolar Disorders, Deventer, The Netherlands; 3 Dimence Mental Health, Center for Mental Health, Almelo, The Netherlands; 4 GGZ Centraal, Center for Mental Health, Hilversum, The Netherlands; 5 IQ Healthcare, Scientific Institute for Quality of healthcare, Nijmegen, The Netherlands; 6 GGZ inGeest, Center for Mental Health, Amsterdam, The Netherlands; 7 Department of Psychiatry, VU University Medical Center, Amsterdam, The Netherlands

**Keywords:** Bipolar disorder, Borderline personality disorder, Randomized controlled trial, STEPPS

## Abstract

**Background:**

Bipolar disorder (BD) and borderline personality disorder (BPD) both are severe and chronic psychiatric disorders. Both disorders have overlapping symptoms, and current research shows that the presence of a BPD has an adverse effect on the course of BD. The limited research available shows an unfavorable illness course, a worse prognosis and response to medication, longer treatment duration, more frequent psychiatric admissions, higher drop-out, increased risk of substance abuse, increased risk of suicide, and more impairment of social and occupational functioning. However, there is no research available on the effect of specific psychotherapeutic treatment for this patients.

**Methods/Design:**

This paper presents the protocol of a RCT to investigate the presence of borderline personality features in patients treated for BD (study part 1) and the effectiveness of STEPPS (Systems Training for Emotional Predictability and Problem Solving) added to treatment as usual (TAU) for BD compared to TAU in patients with BD and comorbid borderline personality features (study part 2). STEPPS is a validated and effective intervention for BPD. The study population consists of patients treated for BD at specialized outpatient clinics for BD in the Netherlands. At first the prevalence of comorbid borderline personality features in outpatients with BD is investigated. Inclusion criteria for study part 2 is defined as having three or more of the DSM-IV-TR diagnostic criteria of BPD, including impulsivity and anger bursts. Primary outcomes will be the frequency and severity of manic and depressive recurrences as well as severity, course and burden of borderline personality features. Secondary outcomes will be quality of life, utilizing mental healthcare and psychopathologic symptoms not primarily related to BD or BPD. Assessment will be at baseline, at the end of the intervention, and at 12 and 18 months follow-up.

**Discussion:**

This will be the first randomized controlled trial of a specific intervention in patients with BD and comorbid BPD or borderline personality features. There are no recommendations in the guideline of treatment of bipolar disorders for patients with this complex comorbidity. We expect that a combined treatment aimed at mood disorder and emotion regulation will improve treatment outcomes for these patients.

## Background

Bipolar disorder (BD) is a complex and heterogeneous mood disorder defined by the occurrence of depressive, manic, hypomanic, and mixed episodes, divided by intervals of longer or shorter duration. DSM-IV-TR [1] classification distinguishes bipolar I disorder (BD-I), bipolar II disorder (BD-II), bipolar disorder Not Otherwise Specified (BD-NOS), and cyclothymia. A study of the World Health Organization [2] reported that BD is associated with significant functional impairment. There are considerable differences in the acute and prophylactic treatment response among patients with BD. Longitudinal studies show that despite treatment there are often recurrent episodes, residual symptoms, cognitive and psychosocial impairments and functional limitations [3]. Many questions regarding the course and outcome of BD and factors that lead to an unfavorable outcome are still open to research. There is evidence that the presence of comorbid personality disorder (PD) has an unfavorable impact on the course of BD [4]. Patients with BD and comorbid PD are more likely to be hospitalized, need more time to achieve symptomatic remission, have more chronic impairments in occupational and social functioning, are less compliant to medication, have greater levels of suicidality, and utilize more psychiatric services than patients with BD without PD [5]. A review reported that BD patients have a significantly higher prevalence of PD than the general population [6]. The prevalence of PD in patients with bipolar disorder is estimated between 30% and 40% [7-10]. This concerns particularly personality disorders from the B and C clusters and in particular borderline personality disorder (BPD). There are considerable areas of clinical overlap between BD and BPD, especially mood instability [11]. In a sample of 375 patients with a bipolar I or II disorder prevalence of BPD was estimated at 37.3% as screened by PDQ-4+, specified as 34,3% in 169 patients who were euthymic at the time of completing the PDQ-4+, 36,8% in 163 depressed patients, and 51.2% in 43 patients with current (hypo-) mania (personal communication R. Kupka: unpublished data from the Stanley Foundation Bipolar Network). Patients who meet diagnostic criteria for both disorders (BD and BPD) have more frequently a history of substance abuse [12]. Furthermore there is evidence that the presence of comorbid PD has a negative impact on the course of BD, leading to more mixed episodes and depressive symptoms, a higher suicide risk, a worse therapeutic response, and less treatment adherence [13]. Given the overlap in clinical presentation of BD and BPD, it has been suggested that there are shared underlying pathophysiological mechanisms [14], [15]. Only three publications report the effectiveness of treatment of BD with comorbid BPD. In a matched case–control study Swartz et al. [16] compared patients who met diagnostic criteria for both BD and BPD with patients with BD without BPD. Both groups received pharmacotherapy and psychotherapy. The results suggest that treatment course may be longer in patients suffering from both BD and BPD. Preston et al. [12] observed that dimensions of BPD improved significantly with lamotrigine treatment. There was a trend for comorbid bipolar patients to require a second psychoactive medication in addition to lamotrigine during extended treatment. Frankenburg and Zanarini [17] compared in a placebo-controlled double-blind study the efficacy of divalproex sodium in the treatment of women with BPD and comorbid BD-II. They found that treatment with divalproex sodium significantly decreased irritability and anger, tempestuousness in relationships, and impulsive aggressiveness.

Currently little research exists on the effects of psychological treatments in patients with BD and comorbid borderline personality features or BPD. There are no specific recommendations included in the guidelines for the treatment of BD in this population (e.g., the Dutch guideline [18]). There clearly is a need to develop new psychotherapies or evaluate existing psychological interventions to improve the course of BD and quality of life as well as reducing mental health care utilization in these patients. This study evaluates the addition of STEPPS (Systems Training for Emotional Predictability and Problem Solving) to treatment as usual (TAU) in patients with BD and comorbid BPD or borderline personality features as described in the inclusion criteria. STEPPS is a cognitive behavioral skills group treatment for BPD developed to improve their emotion regulation. The training consists of 20 weekly sessions of approximately 2,5 hours and has 4 parts: psycho-education, emotion regulation skills, behavioral skills, and emotion handling plan. Two trainers deliver the training. The group size is 8–12 patients. An uncontrolled pilot study presented an indication of the effectiveness of the STEPPS in BPD [19]. A randomized trial [20] showed that BPD patients treated with STEPPS compared with TAU showed larger reduction of BPD symptoms, more quality of life and less general psychological symptoms. All improvements were still significant after 6 months. In another randomized trial [21] STEPPS plus TAU also led to greater improvements in impulsivity, negative affectivity, mood, and global functioning. STEPPS is included in the Dutch guidelines for treatment in BPD [22]. The underlying assumption is that BPD is characterized by a defect in the individual’s internal ability to regulate emotional intensity. The aim of the training is to gain skills to manage emotions and behavior related problems. Friends and family members provide the “reinforcement team” and learn to reinforce and support newly learned skills of the patients (http://www.steppsforbpd.com).

BPD can be conceptualized as a dimensional or spectrum disorder. Patients may suffer from significant symptoms of BPD without meeting the full diagnostic criteria defined by DSM-IV-TR. To increase the clinical relevance of the present study, both patients with BPD as well as those with borderline personality features are included. For this study patients with borderline personality features are included if they have three or more of the DSM-IV-TR diagnostic criteria, with the additional requirement that these include both impulsivity and anger bursts, since STEPPS training is specifically aimed at improving skills to regulate these symptoms.

The study described in this article will investigate the presence of borderline personality features in patients treated for BD (study part 1). Furthermore it will be investigated in a randomised controlled trial if a specific treatment (STEPPS combined with TAU) compared to TAU only will lead to better clinical outcomes as well as an increased quality of life and less care consumptions (study part 2).

## Methods

### Design

#### Study part 1

**Figure 1:**
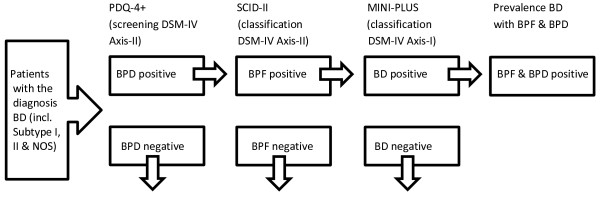
**Flow chart study 1.** BD = bipolar disorder, BPD = borderline personality disorder, BPF = borderline personality features, NOS = not otherwise specified.

Screening for personality disorder with a questionnaire (PDQ4+) in patients with BD treated in a specialized outpatient facility, followed by a semi-structured diagnostic interview (SCID-II) in those patients who screened positive on BPD or borderline personality features relevant for this study. Figure 1 shows the flow chart of study 1.

#### Study part 2

**Figure 2:**
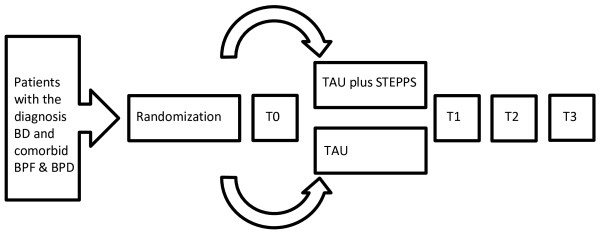
**Flow chart study 2.** BD = bipolar disorder, BPF = borderline personality features, TAU = treatment as usual, STEPPS = Systems Training for Emotional Predictability and Problem Solving.

A multicenter randomized controlled trial in outpatients with BD and comorbid BPD or borderline personality features. The trial will be carried out in specialized outpatient clinics for BD of large mental health institution in the Netherlands. The inclusion period including all follow-up measurements will be one and a half year. The duration of the intervention phase is 20 weeks. Patients will be recruited through treating psychologist, nurses and psychiatrist. Figure 2 shows the participant flow of the RCT.

### Participants

Participants of study part 1 are BD patients aged 18–65 years who are currently receiving treatment in specialized outpatient clinics for BD of large mental health institution in the Netherlands. All participants from study part 1 meet the DSM-IV-TR diagnosis criteria of BD-I, BD-II or BD-NOS, verified by Mini-International Neuropsychiatric Interview – Plus [23]. Respondents who fulfill the criteria for BPD or borderline personality features according to the definition of this study are invited to participate in study part 2. They are approached by letter or are referred by their treating clinicians. Patients receive both written and oral information and signed informed consent, in and exclusion criteria are checked.

### Inclusion criteria

Patients age 18 – 65, diagnosed with BD (BD-I, BD-II and BD-NOS) and comorbid BPD or borderline personality features (at least 3 of 9 DSM-IV-TR criteria for BPD, including both impulsivity and anger bursts).

### Exclusion criteria

Patients with currently severe depressive (score on the Invertory of depressive Symptoms, self-rated version IDS-SR [24] > 38) or manic symptoms (score on the Young Mania Rating Scale YMRS [25] > 20) are excluded from the study. Other exclusion criteria are: currently having a separate treatment for substance abuse; currently receiving a separate psychotherapy; having received STEPPS within the last two years or receiving STEPPS currently; being unable to comply with the STEPPS protocol; and being unable to understand the Dutch language.

### Objectives

#### Study part 1

Primary objective:

• To investigate the prevalence of borderline personality features in outpatients with BD.

#### Study part 2

Primary objectives:

• To investigate the effectiveness of STEPPS added to TAU on the symptoms and course of BD in patients with BD and comorbid borderline personality features.

• To investigate whether addition of STEPPS to TAU is differently associated with a decrease of frequency and severity of manic, hypomanic or depressive episodes.

• To investigate the effectiveness of STEPPS added to TAU on the symptoms, course and burden of borderline personality features.

Secondary objectives:

• To investigate whether addition of STEPPS to TAU is associated with an increase of quality of life.

• To investigate whether addition of STEPPS to TAU is associated with a decrease of utilizing mental healthcare.

• To investigate whether addition of STEPPS to TAU is associated with a decrease of psychiatric symptoms not primarily related to BD or borderline personality features.

### Procedure and outcome measures

Study part 1: The aim of study part 1 is to investigate the prevalence of borderline personality features in patients with a diagnosis of BD. After informed consent all patients with the diagnosis of BD are asked to complete the *Personality Disorder Questionnaire-4+* (PDQ-4+ [26], Dutch translation [27]) to screen for the prevalence of BPD. After screening all patients who score positively on the presence of BPD will be asked to participate in a *Structured Clinical Interview for DSM-IV Personality Disorders* (SCID-II) [28]; Dutch translation [29]. The SCID-II will start with modules concerning BPD. In case of the presence of three BPD symptoms, including both impulsivity and anger bursts, the interview will be continued with modules of other personality disorders to explore further axis-II comorbidity. In case of presence of less than three BPD symptoms (including both impulsivity and anger burst) the interview will be terminated. The diagnosis of BD (inclusive subtype I, II & NOS) will be verified with modules A (depression), C (suicidality) and D (mania) of the MINI-Plus [23]. Modules K & L will be completed to determine substance abuse, and module M part I & II will be completed to exclude schizoaffective disorder. In patients who were previously assessed with the MINI-Plus, a re-test will be completed only if the information is older than 2 years.

**Figure 3:**
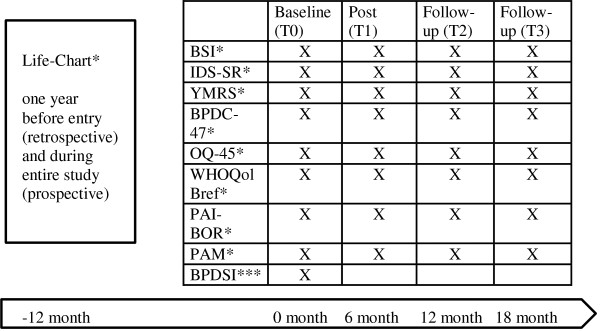
**measurements of study 2.** Note: *self-rated **observer rated ***interview.

Study part 2: After inclusion into the trial patients are randomly assigned either to the STEPPS added to treatment as usual (TAU) for bipolar disorder or the control group receiving TAU for BD only. Patients in the intervention group will be assessed on all outcome measures at baseline (T0), at the end of the intervention (T1) and 12 (T2) and 18 (T3) months after start of treatment. Patients in the TAU control group will be assessed at baseline and at corresponding intervals. The course of BD and utilization of mental healthcare (number of consultations) is measured with the prospective version of the *Life Chart Methodology* (LCM) [30]. At baseline all patients will be assessed with the *Borderline Personality Severity Index* (BPDSI) for severity of borderline symptoms [31], the *Brief Symptom Inventory* (BSI) [32] for severity of general psychological symptoms, the *Inventory of Depressive Symptomatology* (IDS-SR) [24] for severity of depressive symptoms, and the *Young Mania Rating Scale* (YMRS) [25] for severity of manic symptoms. The burden of borderline symptoms will be assessed with the *Borderline Personality Disorder Checklist* (BPDC-47) [33] and *Personality Assessment Inventory – Borderline Features (PAI-BOR)*[34]*.* Quality of life will be measured with the *Outcome Questionnaire* (OQ-45) [35] and *World Health Organization Quality of Life short version* (WHOQol Bref) [36]. Patient empowerment will be assessed with the *Patient Activation Measure* (PAM) [37]. To assess the illness course over the past year, all participants will complete the retrospective version of the LCM. Figure 3 shows a summary of measures of study 2.

### Intervention

Investigational treatment will be STEPPS, as described in the introduction, added to individual TAU (Treatment As Usual) for BD. According to current guidelines for treatment in BD [18] TAU consists of three necessary modules: pharmacotherapy, information and psycho-education, and interventions to improve self-management.

### Sample size

Inclusion of 64 patients for each group has a power of 80% and an alpha of 5% to detect an average difference in Cohen’s d (0.50). Assuming a loss of 10% 140 patients will be enrolled. Patients will participate in STEPPS groups that are normally organized in the participating centers, i.e., there are no groups exclusively set up for this research population. The rationale is that in clinical practice, patients with BD and comorbid borderline personality features will be treated in routinely organized STEPPS groups, together with patients with a primary diagnosis of BPD.

### Statistical analyses

Differences in baseline data between the STEPPS condition and TAU condition are analyzed by t-test for continuous variables (demographic characteristics and scores on the outcome variables). Differences in utilizing mental healthcare between both groups will also be analysed by t-test. Categorical variables will be analyzed by chi-square. Both groups will be compared to an average number of symptomatic days/year, differentiated according to severity (LCM).

Linear Mixed Model (LMM) under the missing at random (MAR) will be used to analyze questionnaires data: the severity of manic and depressive symptoms (IDS-SR & YMRS), severity and course of borderline symptoms (BPDC-47 & PAI-BOR), psychological and somatic distress (BSI total score) and quality of life (OQ-45 & WHOQol Bref).

### Ethical review and trial registration

This RCT has been reviewed and approved by the ethics committee of the VU University Medical Center in Amsterdam, The Netherlands (registration number: 2012/470). It is registered in http://www.trialregister.nl (trial ID: NTR4016).

## Discussion

To our knowledge there is little known about the effectiveness of psychological treatment in patients with BD en comorbid BPD. This will be the first randomized controlled trial to test the efficacy of an integrated treatment consisting of a cognitive behavioral skills training next to TAU in this specific population. The relationship between BD and BPD is still subject of controversy. In a review Paris et al. [38] conclude that BPD and BD may co-occur, but their relationship is not consistent or specific. More knowledge about co-occurrence and comorbidity of these disorders may have an important clinical relevance in order to plan appropriate treatment [39]. It is expected that a psychological treatment aimed at improving emotion regulation will have a positive effect on symptoms of personality disorder as well as BD, and will contribute to an increased quality of life and less care consumption in patients with complex emotional instability.

## Competing interests

The authors have no competing interest in relationship to this study.

## Authors’ contributions

GR is coordinating investigator and grant holder. RK is the principal investigator of this study, involved in the development of the protocol and consultant especially in questions about bipolar disorder. NW is supervisor, involved in the development of the RCT. PG is involved in the development of the protocol and supervises this project. MH is involved in carrying out the study and data collection for study 1. ND was consultant in the development of the study protocol particularly in matters relating to borderline personality disorder. All authors contributed to this paper. All authors read and approved the final manuscript.

## Pre-publication history

The pre-publication history for this paper can be accessed here:

http://www.biomedcentral.com/1471-244X/14/172/prepub
